# Harm avoidance, daily stress, and problematic smartphone use in children and adolescents

**DOI:** 10.3389/fpsyt.2022.962189

**Published:** 2022-09-14

**Authors:** So Young Yoo, Su Mi Park, Chi-Hyun Choi, Sun Ju Chung, Soo-Young Bhang, Jae-Won Kim, Yong-Sil Kweon, Jung-Seok Choi

**Affiliations:** ^1^Department of Psychiatry, SMG-SNU Boramae Medical Center, Seoul, South Korea; ^2^Department of Counseling Psychology, Hannam University, Daejeon, South Korea; ^3^Hanseo Central Hospital, Uijeongbu, South Korea; ^4^Department of Psychiatry, Eulji General Hospital, Seoul, South Korea; ^5^Division of Child and Adolescent Psychiatry, Department of Psychiatry, Seoul National University Hospital, Seoul, South Korea; ^6^Department of Psychiatry, College of Medicine, Uijeongbu St. Mary's Hospital, The Catholic University, Seoul, South Korea; ^7^Department of Psychiatry, Samsung Medical Center, School of Medicine, Sungkyunkwan University, Seoul, South Korea

**Keywords:** problematic smartphone use, stress, harm avoidance, personality, mediation

## Abstract

**Objective:**

Temperament is close to susceptibility to stress and the increased level of stress may lead problematic smartphone use (PSU). In this study, the relationships between harm avoidance (HA) as a personality trait, daily traits, and PSU in children and adolescents were investigated.

**Methods:**

At baseline, all participants (184 children and adolescents, mean age 13.15 years) completed questionnaires on PSU using the Smartphone Addiction Scale-Short Version (SAS-SV) and the Korean Smartphone Addiction Proneness Scale (SAPS). The Daily Hassles Questionnaire (DHQ) and Junior Temperament and Character Inventory (JTCI) were also administered to evaluate stress levels and personality traits. SAS-SV, SAPS, and DHQ were reassessed at 3 and 6 months.

**Results:**

Among JTCI temperament, HA displayed robust positive correlations with SAS-SV, SAPS, and DHQ at all time points. Mediation effects of daily stress on the relationship between HA and PSU were observed at baseline, 3, and 6 months.

**Conclusion:**

The findings suggest that managing stress may important in PSU children and adolescents with high HA.

## Introduction

The number of smartphone users has rapidly increased around the world in recent years (1). For example, the smartphone ownership rate in South Korea was 70.4% in 2019 (2). The drastic increase in smartphone use raises questions about its risks, which include excessive use and psychological problems such as depression, anxiety, and sleep issues (3, 4). Furthermore, smartphone overuse is a risk factor for physical complications such as neck and eye problems (5, 6).

Excessive smartphone use can be categorized as a behavioral addiction, such as gambling disorder and Internet gaming disorder. Presentations commonly associated with addiction, such as preoccupation, loss of control, withdrawal, and functional impairment, have also been found in individuals engaged in excessive smartphone use (7–9). Based on these findings, smartphone addiction is defined as smartphone overuse that results in significant functional impairment. However, the term “problematic smartphone use (PSU)” is also used instead of smartphone addiction because there is insufficient evidence to confirm the existence of smartphone addiction (10). Currently, the terms smartphone addiction and PSU seem to be used synonymously in the literature.v

Children and adolescents are especially prone to PSU, which could be related to immature self-regulation and impulse control (11, 12). In 2019, a nationally representative survey of South Korea showed that 30.2% of adolescents were at risk of smartphone overdependence, which was comparable to 20.0% for all age groups (2). Furthermore, children showed the greatest increase in smartphone overdependence among all age groups (from 20.7% in 2018 to 22.9% in 2019).

Addictive behaviors can be explained by Cloninger's temperament model, which is measured by Temperament and Character Inventory (TCI) (13, 14). According to Cloninger's theory, heritable temperament is composed of the dimension of novelty seeking (NS), harm avoidance (HA), reward dependency (RD), and persistence. NS is related to the dopaminergic system which is responsible for behavioral activation to novel stimulus. On the other hand, HA is related to the system of serotonergic activity and is responsible for inhibiting behavior when encountering or anticipating aversive stimuli. RD relates to rewards in social situations, such as approval from others. Persistence involves in maintenance of a behavior. Among them, high NS and high HA have been found to be associated with PSU as well as other addictive behaviors (15–18).

Temperaments is related to cognitive evaluation of, response to, and selecting coping strategy to stressors (19). In particular, high HA is characterized as fearful, pessimistic, fatigable, and slow to recover from stress, so it has been strongly found in previous studies to be related to stress vulnerability as well as adverse psychological outcomes such as depression and anxiety (20, 21). Stress also plays an important role in PSU. One meta-analysis showed that stress was positively correlated with mobile phone use (22). Furthermore, PSU had a larger association than non-problematic phone use with stress. Academic stress, especially in adolescents, was found to be one of the risk factors for PSU (23).

In this study, we aimed to investigate relationships between HA as a personality trait, levels of stress in a daily life, and PSU in children and adolescents. We hypothesized that individuals with higher levels of stress, and high HA would show positive correlations with PSU. Additionally, we hypothesized that stress would have a mediating effect on the relationship between HA and PSU. We also explored relationships between other temperaments of Cloninger's theory, daily stress, and PSU. In order to examine whether the relationship between the HA, the daily stress and the smartphone use is stable, daily stress and PSU levels were repeatedly measured at baseline, 3 months, and 6 months later. To the best of our knowledge, this is the first study to investigate the mediation effect of daily stress on the relationship between personality traits and PSU in the pediatric population.

## Methods

### Participants

A multicenter clinical cohort study (clinic-cohort for the understanding of Internet addiction rescue factors in early life) was conducted in South Korea from August 2015 to August 2019 to track the natural history of the Internet, gaming, and smartphone addiction in children and adolescents, and identify protective and risk factors. A total of 194 children and adolescents were recruited and screened using the self-report Korean Scale for Internet Addiction for Adolescents (24), Smartphone Addiction Scale-Short Version (SAS-SV) (9). Korean Smartphone Addiction Proneness Scale (SAPS) (25), and the parent-report Internet Addiction Proneness Scale for Children and Adolescents (24). Participants who scored higher than the cut-off value on at least one of these screening questionnaires were enrolled in the cohort study.

The present study included 184 children and adolescents (46 girls; mean age 13.15 years) who had analyzable data for PSU level, stress level, and personality traits. Ten participants who did not respond to any of these questionnaires at baseline were excluded from the study. This study was approved by the Institutional Review Board (IRB) for Human Subjects at the SMG-SNU Boramae Medical Center (IRB No.16-2016-4). Detailed information about the study was provided to the subjects and their parents and written informed consent was obtained before participation.

### Measurements

All participants completed several questionnaires including the SAS-SV, SAPS, Daily Hassles Questionnaire (DHQ), and junior TCI at baseline. PSU and stress levels were reassessed at 3 and 6 months using the SAS-SV, SAPS, and DHQ.

#### Smartphone addiction scale-short version

The SAS is a self-reported questionnaire used to evaluate PSU (9). It comprises 33 items with a six-point Likert scale ranging from 1 (strongly disagree) to 6 (strongly agree). The SAS-SV was developed to screen PSU easily (9). Ten items out of 33 questions were selected by experts. The SAS-SV showed high internal consistency (α = 0.91) and concurrent validity. A total score of 31 or higher in boys and 33 in girls indicated the presence of PSU.

#### Korean smartphone addiction proneness scale

The SAPS was developed to assess PSU in children and adolescents (25). It consists of 15 items with a four-point Likert scale (1 = “strongly disagree,” 2 = “disagree,” 3 = “agree,” 4 = “strongly agree”) across four subdomains: disturbance of adaptive functions, virtual life orientation, withdrawal, and tolerance. Items related to virtual life orientation subdomain are SAPS-specific features. Higher scores reflect a greater risk of PSU. The SAPS was shown to be both valid and reliable (α = 0.88). A score of 42 or higher in the SAPS indicated the presence of PSU.

#### Junior temperament and character inventory

The JTCI was developed to assess temperaments and character traits in adolescents (26, 27). Based on Cloninger's biosocial model of personality (13, 14), it consists of four temperament dimensions (novelty seeking, harm avoidance, reward dependence, and persistence) and three-character trait dimensions (self-directedness, cooperativeness, and self-transcendence). In this study, we used norm-referenced T-scores of temperament dimensions.

#### Daily Hassles Questionnaire

The DHQ is a well-validated questionnaire to measure daily life stresses related to factors such as parents, family, friends, academics, and school (28). It was modified and validated for adolescents in Korea (29). The Korean version of the DHQ consists of 36 items with a four-point Likert scale. Higher scores indicate greater stress levels. The internal consistency of the DHQ in this study was 0.934.

### Statistical analyses

Descriptive statistics included age, gender, stress level, PSU level, and temperament variables at baseline. Relationships between variables were examined using Pearson's correlation analysis. Mediation model analyses were performed using the PROCESS macro with 5,000 bias-corrected bootstrap samples (30). All analyses were conducted using IBM SPSS Statistics version 28.0 (SPSS Inc., Chicago, IL, USA), and statistical significance was set at *p* < 0.01 for correlation analyses and <0.05 for mediation models.

## Results

### Demographic and clinical characteristics

**Figure 1:**
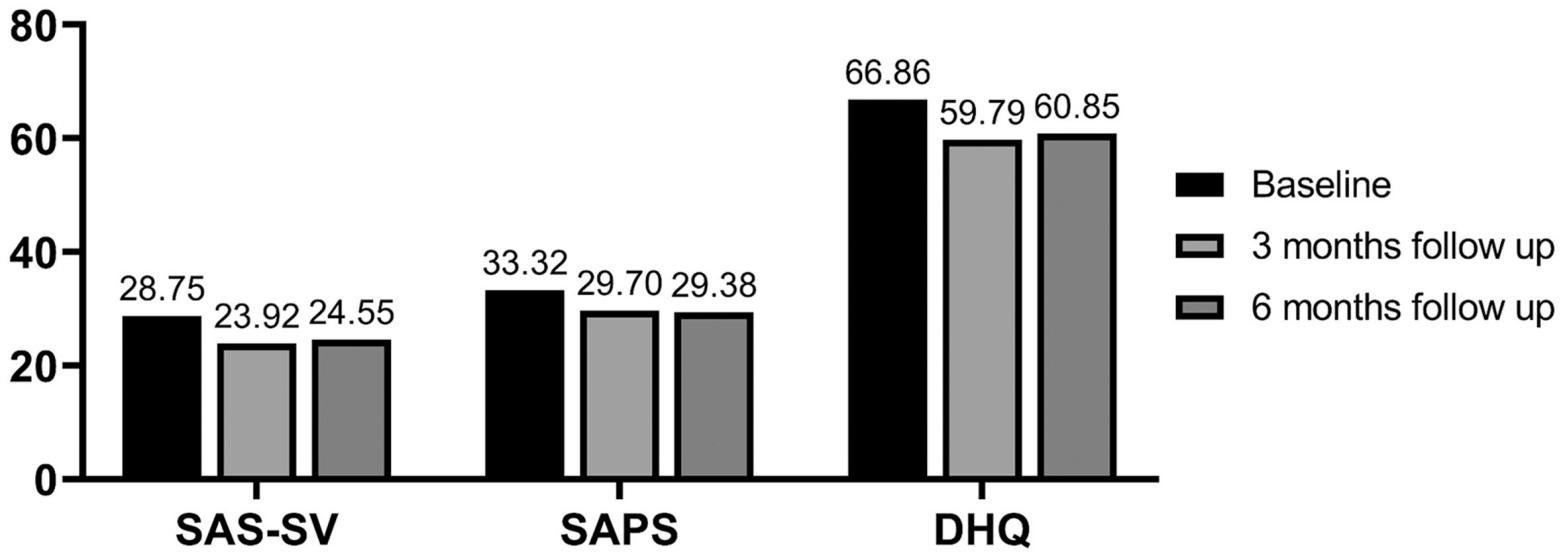
Descriptive statistics of problematic smartphone use and stress level. SAS-SV, Smartphone Addiction Scale-Short Version; SAPS, Korean Smartphone Addiction Proneness Scale; DHQ, Daily Hassles Questionnaire.

In this study, we included 184 children and adolescents aged 7–18 years (Table 1). The majority of the participants were boys (75.0%). Scores of the smartphone addiction scales ranged from 10 to 56 and 15 to 55 in the SAS-SV and SAPS, respectively. The scores of the DHQ ranged from 36 to 123. The mean scores of PSU and stress level at baseline, 3 months, and 6 months are presented in Figure 1.

**Table 1:** Demographics and clinical characteristics (*N* = 184).

**Variables**	**M or N**	**SD**
Age (years)	13.15	2.48
Sex (male, %)	138 (75.0%)	
Developmental period (adolescents, %)	135 (73.4%)	
SAS-SV	28.75	12.09
SAPS	33.32	8.88
DHQ	66.86	18.29
JTCI		
NS	50.99	10.74
HA	49.56	12.02
RD	45.89	10.36
P	47.45	9.67
Comorbid diagnosis		
Depression	31 (16.8%)	
Anxiety	17 (9.2%)	
ADHD	56 (30.4%)	
Other	1 (0.5%)	

SAS-SV, Smartphone Addiction Scale-Short Version; SAPS, Korean Smartphone Addiction Proneness Scale; DHQ, Daily Hassles Questionnaire; JTCI, Junior Temperament Character Inventory; NS, Novelty Seeking; HA, Harm Avoidance; RD, Reward Dependence; P, Persistence; ADHD, attention deficit hyperactivity disorder.

### Correlation between personality trait, daily stress, and PSU

Table 2 show correlational results of variables at each time point of baseline, 3 months, and 6 months. All PSU levels at all time points were positively correlated with each other and daily life stress levels at all time points were positively related to PSU. Among the temperament dimensions of the JTCI, only HA robustly showed positive correlations with the SAS-SV, SAPS, and DHQ at all time points (all *p*s < 0.01). NS showed positive correlations with the SAS-SV, SAPS, and DHQ at baseline, but did not at 3 and 6 months. RD was not correlated to any PSU or stress-related variables. Therefore, we used only HA among JTCI in further mediational analyses.

**Table 2:** Correlation between JTCI temperament, PSU, and daily Stress.

		**1**	**2**	**3**	**4**	**5**	**6**	**7**	**8**	**9**	**10**	**11**	**12**	**13**
Baseline	1.NS	1												
	2.HA	0.273^**^	1											
	3.RD	−0.053	−0.058	1										
	4.P	−0.182	−0.127	0.318^***^	1									
	5.SAS-SV	0.253^**^	0.329^***^	0.054	−0.143	1								
	6.SAPS	0.249^**^	0.333^***^	−0.014	−0.227^**^	0.864^***^	1							
	7.DHQ	0.211^**^	0.403^***^	−0.100	−0.204^**^	0.293^***^	0.336^***^	1						
3 Months	8.SAS-SV	0.117	0.305^***^	0.081	−0.258^**^	0.607^***^	0.557^***^	0.265^**^	1					
	9.SAPS	0.136	0.289^***^	0.057	−0.241^**^	0.575^***^	0.603^***^	0.240^**^	0.896^***^	1				
	10.DHQ	0.194	0.405^***^	−0.090	−0.089	0.251^**^	0.283^**^	0.614^***^	0.350^***^	0.368^***^	1			
6 Months	11.SAS-SV	0.099	0.395^***^	0.051	−0.157	0.619^***^	0.561^***^	0.355^***^	0.724^***^	0.703^***^	0.383^***^	1		
	12.SAPS	0.106	0.388^***^	0.005	−0.251^**^	0.538^***^	0.574^***^	0.418**^*^	0.660^***^	0.725^***^	0.449^***^	0.871^***^	1	
	13.DHQ	−0.081	0.270^**^	−0.091	0.018	0.201	0.230	0.468^***^	0.339^***^	0.287^**^	0.552^***^	0.348^***^	0.315^***^	1

** P < 0.01,

*** P < 0.001. JTCI, Junior Temperament Character Inventory; NS, Novelty Seeking; HA, Harm Avoidance; RD, Reward Dependence; P, persistence; PSU, Problematic Smartphone Use; SAS-SV, Smartphone Addiction Scale-Short Version; SAPS, Korean Smartphone Addiction Proneness Scale; DHQ, Daily Hassles Questionnaire.

### Mediation effects of stress on the relationship between HA and PSU

**Figure 2:**
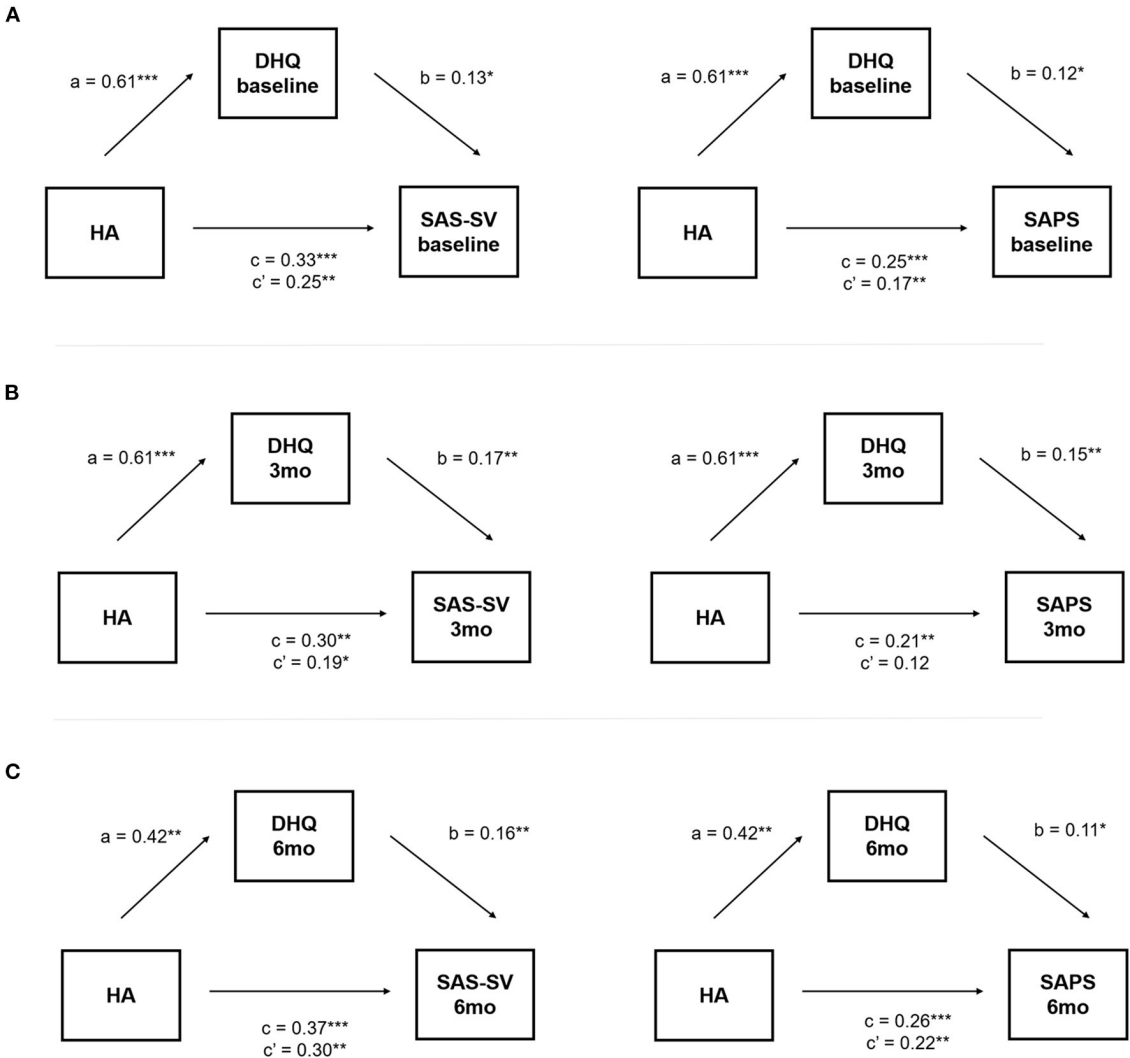
Mediation effects of daily stress on the relationship between HA and PSU at each time point. **(A)** represents results at baseline, **(B)** at 3 months, and **(C)** at 6 months. Unstandardized coefficients (β) are presented. a is effect of HA on daily stress; b is effect of daily stress on PSU; c is total effect of HA on PSU; c' is direct effect of HA on PSU. *P < 0.05, **P < 0.01, ***P < 0.001. HA, Harm Avoidance, PSU, Problematic Smartphone Use, SAS-SV, Smartphone Addiction Scale-Short Version; SAPS, Korean Smartphone Addiction Proneness Scale; DHQ, Daily Hassles Questionnaire.

We found significant indirect effects of daily stress on the relationship between HA and PSU at all time points (Table 3). At baseline, the effects of HA on PSU measured by SAS-SV and SAPS were partially mediated by baseline stress levels (Figure 2A). At 3 months, the baseline HA was partially mediated on the way of SAS-SV and fully mediated of SAPS by daily stress at that time point (Figure 2B). At 6 months, the effects of HA on both SAS-SV and SAPS were partially mediated by the stress (Figure 2C).

**Table 3:** Bootstrapped indirect effects of daily stress on the relationship between HA and PSU.

	**Paths**	**Boot Indirect effect**	**Boot SE**	**LLCI**	**ULCI**
Baseline	HA → DHQ → SAS-SV	0.0773	0.0365	0.0101	0.1549
	HA → DHQ → SAPS	0.0734	0.0275	0.0239	0.1323
3 Months	HA → DHQ → SAS-SV	0.1061	0.0440	0.0317	0.2087
	HA → DHQ → SAPS	0.0902	0.0333	0.0338	0.1686
6 Months	HA → DHQ → SAS-SV	0.0684	0.0389	0.0131	0.1702
	HA → DHQ → SAPS	0.0441	0.0265	0.0085	0.1181

HA, Harm Avoidance; PSU, Problematic Smartphone Use; SAS-SV, Smartphone Addiction Scale-Short Version; SAPS, Korean Smartphone Addiction Proneness Scale; DHQ, Daily Hassles Questionnaire; SE, standard error; LLCI, lower level confidence interval; ULCI, upper level confidence interval.

## Discussion

In this study, we examined relationships between personality traits, level of stress, and PSU in children and adolescents with a prospective design. As the hypothesis for the mediation effect of daily stress, the relationships between HA and PSU were mediated by stress throughout 6 months of follow-up. Among the JTCI temperament dimensions, only HA was consistently related to both daily stress and PSU while NS, RD, and persistence did not.

HA represents the tendency to respond intensely to aversive stimuli and is related to behavioral inhibition and serotonergic activity (14, 31). Previous studies have reported that high HA is a risk factor for PSU in adolescents and adults (32, 33). In addition, neuroticism of the Big Five personality which shares anxiety-related traits with HA has been found be positively associated with PSU (34, 35). Neuroticism refers to a dimension of nervousness and negative emotionality (36). Smartphone use and anxiety are positively correlated and this association is larger in PSU than in non-problematic use in adults (22). Consistent with previous studies, we found that among children and adolescents, levels of PSU and HA are positively correlated.

Individuals with high HA are likely to show strong reactions and attentional biases toward stressors (13). High HA is related with increased stress, cortisol level, maladaptive coping style, and maladaptive emotional regulation as well as depression and anxiety (37–39). Those with high HA might inevitably experience more stress in their daily life. To relieve negative emotions caused by stress, children and adolescents with HA tend to use smartphones excessively as a coping strategy (23, 40). Our findings can be explained with findings support the “Interaction of Person-Affect-Cognition-Execution” model, which is a theoretical framework to explain the process underlying an addictive behavior of Internet use (41). According to this model, specific personality traits result in affective and cognitive responses to situational triggers, and lead to addictive behavior. This finding suggests that stress plays an important role in developing and maintaining PSU among children and adolescents with high HA.

This study focused on the effect of HA and daily stress on PSU, which were found to be stable at all time points. We measured PSU and daily stress level during follow up. Thus, it is possible that the effect of the reaction tendency appearing at one time point was excluded. We did not focus on changes in the levels of daily stress and PSU within individuals, so caution is needed in interpretation. Nevertheles, the result suggests that HA could be associated with clinical conditions such as depression and anxiety as well as daily stress. According to the findings of this research team in previous internet addiction research, the behavioral inhibition system affected internet addiction through depression and anxiety, whereas the behavioral activation system closely related to NS influenced internat addiction through impulsivity as a mediator (42). Alternative explanations may be raised, such as PSU lowers stress tolerance and increase perceived stress level. The results of this study call for future study to invesigate whether managing stress in daily life may be helpful to PSU children and adolescents. For example, through non-pharmacological interventions such as cognitive behavioral therapy, it is also possible to improve various coping skills and emotional regulation skills in daily life.

This study has some limitations. First, in this study, we included only those who exceeded the cut-off values on screening questionnaires, which limits generalizing our findings to non-clinical populations. Second, because of the relatively small number of women and children, it was limited to thoroughly analyze the differences according to gender and school age. Enlarging sample with more female and children subjects is needed to become representative. Third, the result may reflect the cultural specificity of South Korea (i.e., high smartphone penetration). Fourth, levels of HA and stress were not measured by biological markers, such as functional imaging or hormones that may further explain the mechanism. Therefore, further studies with a control group and biological markers are needed to confirm our findings.

In summary, this study highlighted the role of daily stress for children and adolescents with PSU. The association between HA and PSU is mediated by the level of daily stress. This finding implies that stress management may be an important for the treatment of PSU in children and adolescents with high HA trait.

## Data availability statement

The original contributions presented in the study are included in the article/supplementary materials, further inquiries about data access can be directed to the corresponding authors.

## Ethics statement

The studies involving human participants were reviewed and approved by the Institutional Review Board (IRB) for Human Subjects at the SMG-SNU Boramae Medical Center (IRB No.16-2016-4). Written informed consent to participate in this study was provided by the participants' legal guardian/next of kin.

## Author contributions

S-YB, Y-SK, and J-SC created and organized the study and collected the data. SY, SP, C-HC, and SC performed analysis and interpreted the results. J-WK and J-SC supervised the statistical analysis and contributed to the interpretation of data. SY and SP wrote the first draft of the manuscript. All authors critically reviewed and approved the final version of the manuscript.

## Funding

This research was funded by the Korea Healthcare Technology R&D Project, Ministry for Health and Welfare, Republic of Korea (HM14C2603 to Y-SK), Korea Mental Health R&D Project, funded by the Ministry of Health and Welfare, Republic of Korea (HI22C0404 to J-SC), and a grant from the National Research Foundation of Korea (Grant No. 2021R1F1A1046081 to J-SC).

## Conflict of interest

The authors declare that the research was conducted in the absence of any commercial or financial relationships that could be construed as a potential conflict of interest.

## Publisher's note

All claims expressed in this article are solely those of the authors and do not necessarily represent those of their affiliated organizations, or those of the publisher, the editors and the reviewers. Any product that may be evaluated in this article, or claim that may be made by its manufacturer, is not guaranteed or endorsed by the publisher.
